# Longitudinal Antibody Dynamics Following SARS-CoV-2 Viral-Vectored and mRNA Booster Vaccination in Ghanaian Adults

**DOI:** 10.3390/vaccines14040303

**Published:** 2026-03-28

**Authors:** Frederica D. Partey, Hidaya Mohammed, Frank Osei, Abigail Naa Adjorkor Pobee, Doris E. Atta-Poku, Yvette A. Ansah, Mary M. A. K. Owusu-Amponsah, Nana Yaa A. Appiah, Nana Akua O. Koranteng, Esther Appiagyei-Mintah, Theophilus Brenko, Stella Nartey, Peter K. Quashie, Michael F. Ofori, Kwadwo A. Kusi

**Affiliations:** 1 Department of Immunology, Noguchi Memorial Institute for Medical Research, College of Health Sciences, University of Ghana, Accra P.O. Box LG 581, Ghananyappiah@noguchi.ug.edu.gh (N.Y.A.A.); tbrenko@noguchi.ug.edu.gh (T.B.); mofori@noguchi.ug.edu.gh (M.F.O.); 2 Department of Microbiology and Immunology, School of Medical Sciences, University of Cape Coast, Cape Coast P.O. Box 24, Ghana; 3 West Africa Centre for Cell Biology of Infectious Pathogens, Department of Biochemistry, Cell and Molecular Biology, College of Basic and Applied Sciences, University of Ghana, Accra P.O. Box LG 54, Ghana; pquashie@ug.edu.gh

**Keywords:** COVID-19 vaccination, heterologous and homologous booster vaccination, COVID-19 booster vaccination sub-Saharan Africa, longitudinal antibody kinetics

## Abstract

**Background/objectives**: SARS-CoV-2 antibodies wane after natural infections and vaccinations. COVID-19 booster vaccination enhances the durability and functionality of antibodies against emerging SARS-CoV-2 variants. Data on booster-induced antibody durability in sub-Saharan Africa remain sparse. Comparative analysis of vaccine-induced responses between heterologous and homologous vaccination regimens remains limited. This study evaluated longitudinal RBD-specific IgG responses following homologous and heterologous COVID-19 booster vaccination in previously vaccinated adults. **Methods**: Adults with prior mRNA or adenoviral-vectored vaccination were boosted with either Pfizer (mRNA) or Janssen (adenoviral-vectored) vaccines. Plasma IgG binding to Wuhan, Delta, and Omicron RBDs was measured pre-booster and at 3, 6, and 9 months. A total of 181 participants were enrolled between November 2022 and October 2023. **Results**: More than 60% of participants had detectable pre-booster RBD- and N-antigen-specific IgG. Booster vaccination substantially increased Wuhan-specific RBD-IgG at three months, with limited boosting of Delta and Omicron responses. Antibody levels waned to pre-booster concentrations by month nine. Heterologous boosting with a viral-vectored prime followed by Pfizer mRNA significantly enhanced both peak RBD-IgG levels and durability. **Conclusions**: These longitudinal data provide rare real-world evidence on booster immunogenicity in African adults and demonstrate that heterologous regimens confer a short- to intermediate-term advantage in antibody magnitude compared to a homologous regimen. This benefit was most pronounced within the first six months post-boost. The findings support additional booster dosing to strengthen protection against emerging variants in sub-Saharan Africa.

## 1. Introduction

Immunity against SARS-CoV-2 wanes over time after both natural infection and vaccination. COVID19-booster vaccinations are recommended to maintain long-term immunity and mitigate the emergence of immune-evasive SARS-CoV-2 variants. Several studies in developed countries have demonstrated that booster vaccinations enhance vaccine-induced immune responses against emerging variants, resulting in extended maintenance of protective immunity [1,2,3]. However, in sub-Saharan Africa, there is a paucity of real-world data demonstrating the dynamics and durability of additional COVID-19 booster vaccinations, particularly after the emergence of the Omicron variant and its sub-lineages [4,5,6]. Addressing this gap is essential, as immune kinetics derived largely from high-income country cohorts may not be directly generalizable to African populations that differ in baseline immune activation, infection exposure histories, and vaccine rollout contexts [7]. These insights are invaluable as the continent manages COVID-19 alongside other infectious diseases and efforts towards future pandemic preparedness and prevention [8].

In Africa, the COVID-19 pandemic followed a different course than that in high-income countries. COVID-19 infections in sub-Saharan Africa were largely asymptomatic [9], although serosurveys have reported high exposure [10,11,12]. COVID-19 vaccines reduce the severity, morbidity, and mortality of the disease in infected individuals. Africa’s late roll out of COVID-19 vaccines is attributed to several factors, including vaccine nationalism [8], vaccine hesitancy within the population, and politics. By the end of 2023, 440 million individuals are estimated to have received at least one dose of the COVID-19 vaccine in the WHO African region [13]. Like other sub-Saharan countries, Ghana initially received COVID-19 vaccines from the COVAX facility in 2021. As of December 2023, approximately 15 million Ghanaians had received at least one dose of the COVID-19 vaccine, and 7 million had been administered booster vaccinations [14]. Vaccinations were carried out in phases as vaccines became available. The vaccines administered included viral-vectored (AstraZeneca, Janssen, and Sputnik V) and mRNA-based vaccines (Pfizer-BioNTech and Moderna).

During the pandemic, SARS-CoV-2 evolved with the emergence of several immune-evasive variants. Notable among the variants was the Omicron variant that emerged in late 2021 [15]. The Omicron variant is highly mutated and transmissible but less virulent compared to previous variants [16]. However, even in countries with significantly high vaccine coverage, there was a surge in COVID-19 cases among vaccinated individuals and increased hospitalizations. There were concerns that the high transmissibility of the Omicron variant could overwhelm global healthcare systems which had been stretched since the pandemic began [17]. Thus, many countries launched booster vaccination campaigns to administer additional vaccine shots, particularly to high-risk groups such as the elderly and immunocompromised. Immunological studies in different populations from high-income countries show a slower decay of viral spike protein-directed antibodies against the prototypical Wuhan (WH) strain in persons receiving at least three vaccine doses compared to those with two vaccine doses [1,2]. Despite the increased half-life of serum-binding and neutralizing antibody levels against the WH, binding and neutralizing antibodies against other variants particularly, Omicron sub lineages were significantly lower [2]. This necessitated the update of COVID-19 vaccine design with sequences from emerging immune evasive variants. Examining long-term dynamics of vaccine-induced humoral and cellular responses in different populations is critical to inform development of vaccines with improved efficacy and is useful for future pandemic preparedness such as timing of vaccination. Real-world kinetics of COVID-19 vaccine-induced immunity in sub-Saharan African studies has mainly focused on individuals who received one or two vaccine doses in the primary vaccination [5,6]. We previously examined the induction of cross-reactive binding and neutralizing antibody levels between naturally infected and previously exposed vaccinated individuals [18]. Within a cohort of Ghanaian adults, we established that vaccination led to higher levels of SARS-CoV-2 binding and neutralizing antibodies than natural infection alone. In this study, we examined the kinetics of spike protein-specific antibody responses in fully vaccinated individuals who received additional booster doses over a period of 9 months. We determined the durability and cross-reactivity of COVID-19 vaccine-induced antibody responses in previously vaccinated Ghanaian adults after booster immunization with the Pfizer BioNTech BNT162b2 mRNA vaccine (Pfizer) or Ad26.COV2.S viral-vectored vaccine (Janssen).

## 2. Materials and Methods

### 2.1. Ethics Statement

Ethical approval for the study was sought from the Noguchi Memorial Institute for Medical Research Institutional Review Board (NMIMR-IRB, approval number CPN 010/22-23), after obtaining scientific approval from the NMIMR Scientific and Technical Committee. Each study participant provided written informed consent prior to their inclusion in the study. All clinical and experimental procedures were conducted in compliance with the principles of the Belmont Report and the guidelines of the Declaration of Helsinki.

### 2.2. Study Design and Population

This longitudinal study enrolled 181 individuals aged ≥18 years who had previously received at least one dose of a COVID-19 vaccine. The study was conducted between November 2022 and November 2023 during a vaccination drive in Ayawaso West Municipal district in Accra, the capital of Ghana. The vaccination campaign primarily aimed to administer booster doses to previously vaccinated individuals. Before the study participants received the booster vaccine, venous blood samples were drawn from them into heparinised tubes. After receiving the booster doses, the participants were followed up for 3, 6, and 9 months and venous blood samples were collected at each follow-up visit. All blood samples were centrifuged at 1500× *g* for 10 min to separate the plasma. The separated plasma was stored at −20 °C or lower until further use. The COVID-19 vaccine types administered as boosters to the study participants were the Pfizer BioNtech BNT162b2 mRNA vaccine (Pfizer Michigan, MI, USA) and Janssen Ad26.COV2.S viral vector vaccine (Janssen, Baltimore, MD, USA ).

### 2.3. SARS-CoV-2 Antigens

The nucleocapsid (N) protein was expressed as previously described [19]. The spike receptor-binding domain (RBD) protein of the Wuhan strain was expressed as previously described [20]. RBD from Delta (# 40592-V08H115) and Omicron (# 40592-V08H143), expressed with a polyhistidine tag at the C-terminus in HEK293 cells, were sourced from Sino Biological (Pennsylvania, PA, USA).

### 2.4. Plasma IgG Reactivity ELISA

Plasma SARS-CoV-2-specific IgG levels were estimated using an indirect ELISA method, as previously described [21]. Briefly, 96-well Nunc Maxisorp plates were coated with SARS-CoV-2 antigens and incubated at 4 °C overnight. RBD antigens from the three different viral strains were coated at 2 µg/mL and N protein was coated at 0.5 µg/mL. The plates were then washed and blocked with PBS/BSA for 1 hat RT. After blocking, plasma samples were diluted 1:100 and added to the wells in duplicate. This dilution was selected based on prior optimization experiments performed in our previous study [18] using the same ELISA protocols. Pre-pandemic samples (*n* = 13) collected in 2018 were used as negative controls and the COVID-19 convalescent plasma pool served as positive control. COVID-19 convalescent plasma used as a positive control was prepared as a pooled plasma sample from individuals (*n* = 10) with PCR-confirmed SARS-CoV-2 infection collected between June and December 2021, during a period when the Delta variant predominated. This standardized plasma pool was aliquoted, stored at −20 °C and used consistently across all ELISA assays to minimize inter-assay variability. The pre-pandemic samples were collected within the same population in the Ayawaso West Municipal district in Accra, the capital of Ghana. Both control samples were plated at the same dilution as test samples. Following sample incubation, the plates were washed, and rabbit anti-human secondary antibody was added to the plates for 1 h. Subsequently, the plates were washed, and IgG binding was detected by incubation with 3,3′,5,5′-tetramethylbenzidine (TMB) for 15 min at RT. Color development was stopped by the addition of 2 M H_2_SO_4_ and the optical density (OD) was measured at 450 nm using a Biotek plate reader. Antibody levels are presented as normalized OD calculated as (OD_test_ − OD_blank_)/(OD_positive control_ − OD_blank_). The cutoff for seropositivity was defined as normalized ELISA OD + (3 × mean standard deviations) of the pre-pandemic plasma samples. IgG reactivity against the ancestral Wuhan variant was evaluated in the entire study cohort (n = 181). Due to limited sample availability, serological assessment against the Delta and Omicron variants was restricted to a subset of 45 participants.

### 2.5. Data and Statistical Analysis

Summary statistics were reported as median and range, and categorical variables were presented as frequencies. Chi-square (χ^2^) test was used for comparison of frequencies. The Kruskal–Wallis test was used to compare IgG levels across different timepoints and where required, post hoc pairwise comparisons were conducted using the Benjamini–Hochberg-adjusted Wilcoxon test. At each timepoint, the Mann–Whitney test was used to compare IgG levels before and after vaccination between and within different participant groups. All comparisons were two-tailed and *p* values < 0.05 were regarded as statistically significant. Data analyses were performed using GraphPad Prism software (version 10.0).

## 3. Results

### 3.1. Pre-Booster Characteristics

The study analyzed samples from 181 adults with a median age of 23 years (range 18–68 years) from the Ayawaso West Municipal District in Accra, Ghana, who were recruited as part of the Immunocov study. Of these, 36.2% were female. All participants had previously received full vaccination with either a single or double dose vaccine against COVID-19, and our analysis focused on antibody dynamics after booster vaccination among our participants (Table 1). Of the 181 individuals, 27.6%, 63.5%, and 8.8% received one, two, and three vaccine doses prior to their inclusion in this study and the subsequent administration of the booster shot. For their previous full vaccination, participants received different COVID-19 vaccines, including the Pfizer, AstraZeneca, Janssen, and Moderna COVID-19 vaccines.

Previously vaccinated adults were recruited in Accra, Ghana, and given either an mRNA or viral vector-based booster vaccination. Homologous vaccination in this study refers to the use of same vaccine platform (mRNA or viral vector) in both prime and booster vaccinations. Heterologous vaccination refers to the use of different vaccine platforms in prime and booster vaccination (mRNA prime/viral boost or viral prime/mRNA boost). N refers to number of participants in each catergory.

### 3.2. SARS-CoV-2 Antibody Levels in Participants’ Plasma Before and After Booster Administration Pre-Booster

**Figure 1:**
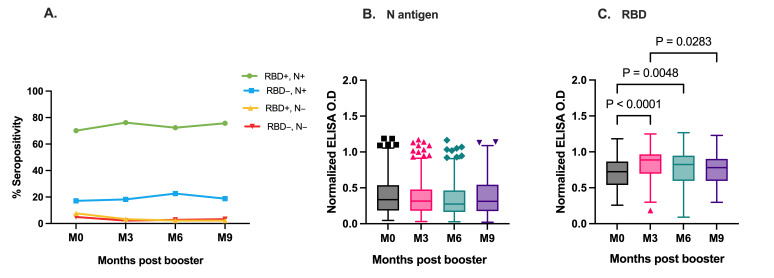
Plasma RBD- and N-specific IgG levels before and after booster vaccination. (**A**) Percentage seroprevalence before and after booster vaccination: individuals seropositive for both RBD and N antigens, RBD+, N+; seropositive for N antigen only RBD−, N+; seropositive for RBD only RBD+, N−; and seronegative for RBD and N antigens, RBD−, N−. Box plots showing IgG levels against N antigen (**B**) and RBD (**C**) across sampling time points. The box plot represents the interquartile range, the horizontal line indicates the median, and the whiskers represent 1.5 × the interquartile range. M0, 3, 6, 9 indicate pre-booster and months 3, 6, and 9 post-boost, respectively.

First, we measured plasma RBD- and N-specific IgG levels against the Wuhan strain before booster vaccination to determine the pre-booster SARS-CoV-2 antibody levels. While spike and RBD-specific IgG levels are expected to be boosted by the vaccines, N-specific IgG levels can only be boosted by natural virus exposure and not vaccination; hence, plasma N antigen levels were used as a proxy for SARS-CoV-2 natural exposure. Of the 181 participants, 127 (70.17%) had detectable IgG levels against both N- and RBD antigens (Figure 1A) pre-booster before receiving a booster shot. Of the remaining participants, 14 (7.73%) were seropositive for only N-specific IgG, and 31 (17.12%) were seropositive for only RBD-specific IgG. Only nine (4.97%) individuals were seronegative for both N- and RBD-specific IgG. Overall, the median plasma N-antigen-specific IgG levels were steady over the 9-month period (*p* = 0.52) (Figure 1B). In contrast, there was a marked increase in plasma RBD-specific IgG levels three months after the booster vaccination (Figure 1C). The relative RBD-specific IgG level (normalized to positive control) increased from 0.73 pre-booster to 0.89 3 months after booster administration (Figure 1C, *p* < 0.0001). RBD-specific IgG levels were sustained for up to 6 months. We observed a significant decrease in RBD-specific IgG levels at month 9 compared with that at month 3. Based on the participants’ pre-booster serostatus, we compared RBD-specific IgG levels in vaccinated participants at months 3, 6 and 9. The thick horizontal line represents the median and the error bars represent the interquartile range.

### 3.3. RBD Antibody Levels After Booster Vaccination Correlate with Number of Vaccine Doses Previously Received

**Figure 2:**
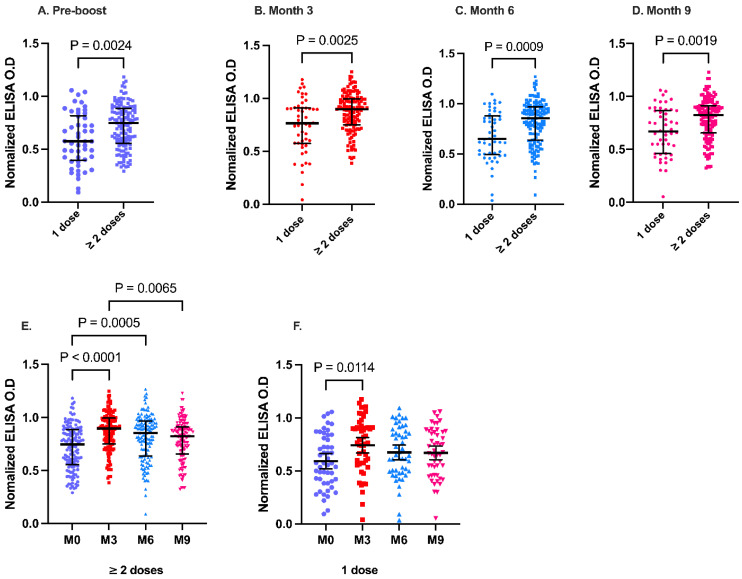
RBD IgG antibody kinetics between recipients of single (n = 50) and two or more vaccine doses (n = 131) before and after the booster vaccination. Median RBD-IgG levels based on the number of previous vaccine doses at pre-booster (**A**), months 3 (**B**), 6 (**C**), and 9 (**D**). Booster antibody durability over 9 months among double-dose (**E**) and single-dose (**F**).

Before receiving booster vaccinations, some study participants had previously received only a single vaccine dose, whereas others had received double vaccine doses. The participants who received a single vaccine dose during primary vaccination were mainly recipients of the Janssen vaccine. During the initial phase of the vaccination campaign, the Ghana Health Service administered the Janssen vaccine as a single vaccine dose in line with the manufacturer recommendations. All other vaccines were administered as double-dose vaccines. Thus, we compared the antibody levels between individuals who received a single dose and those who received at least two doses of the vaccine before the present study (Figure 2). Individuals who received at least two vaccine doses prior to receiving the booster had significantly higher anti-RBD IgG levels at the pre-booster. Antibody levels remained significantly higher at 3, 6, and 9 months post booster vaccination in recipients of two or more vaccine doses than in the single-dose recipients (Figure 2A–D). Overall, there was a decline in anti-RBD IgG levels after three months in both groups. However, the administration of a booster dose significantly enhanced the durability of anti-RBD IgG levels in individuals who received two prior vaccine doses (Figure 2E). We observed significantly higher RBD IgG levels at months 3 (*p* < 0.0001) and 6 months than at pre-booster (*p* = 0.0005). Comparatively, in individuals who only received a single vaccine dose before booster, the Anti-RBD IgG levels declined after month 3 compared to pre-booster levels (Figure 2F).

### 3.4. SARS-CoV-2 Variant Cross-Reactive Antibody Levels at Pre-Booster and After Booster Vaccination

**Figure 3:**
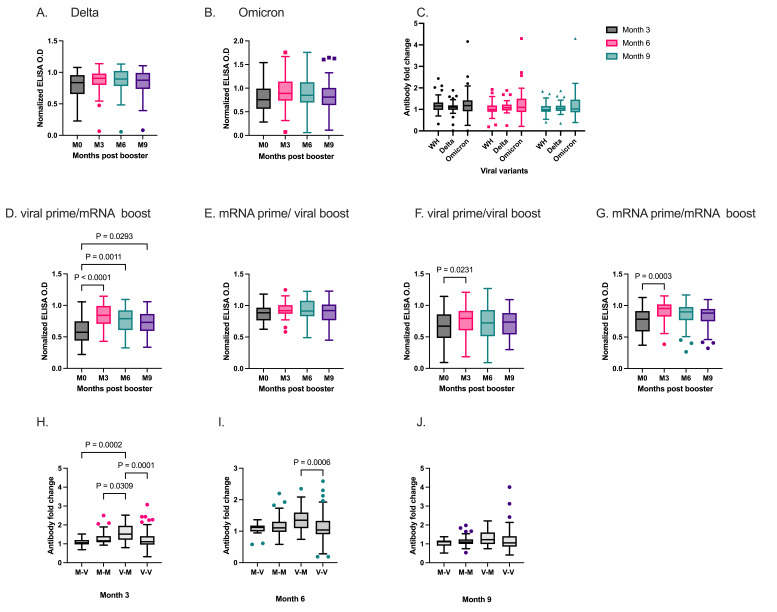
Cross-reactive RBD antibody levels before and after booster vaccination. RBD-specific IgG levels against Delta (**A**) and Omicron (**B**) at pre-booster and 3, 6, and 9 months after booster administration. Comparison of RBD-IgG antibody fold change against Wuhan (WH), Delta, and Omicron at months 3, 6, and 9 (**C**). Comparative analysis of RBD IgG levels against Wuhan strain between heterologous (**D**,**E**) and homologous (**F**,**G**) booster vaccination. Antibody fold change between heterologous and homologous at month 3 (**H**), month 6 (**I**) and month 9 (**J**). Box plots represent the median and the interquartile range. The whiskers represent 1.5 times the interquartile range. M-V; mRNA prime/viral boost (*n* = 20), M-M; mRNA prime/mRNA boost (*n* = 45), V-M; viral prime/mRNA boost (*n* = 47), V-V; viral prime/viral boost (*n* = 69).

Booster doses are recommended to enhance the immunity against emerging SARS-CoV-2 variants. Thus, we examined the levels of cross-variant IgG against RBD from Delta and Omicron variants in a subset of our cohort, *n* = 45 (Figure 3A,B) before and after booster vaccination. The levels of RBD-specific IgG against Delta and Omicron variants increased marginally, albeit insignificantly after booster vaccination. The antibody fold change against Delta and Omicron was compared to that against the Wuhan strain at each time point post-booster. There was no difference at month 3 between the wild-type and Delta RBD IgG levels (Figure 3C).

Next, we stratified the participants according to whether they had received the same vaccine type in the primary and booster series vaccinations. Based on the vaccines received, four groups were formed (Figure 3D–G). Heterologous vaccination was defined as the receipt of viral vector vaccines during primary vaccination and mRNA-based vaccines during booster vaccination, and vice versa. Heterologous vaccination (viral prime/mRNA boost) (Figure 3D) and homologous (Figure 3F,G) booster vaccination led to a significant rise in RBD IgG levels three months post-vaccination. In the case of heterologous vaccination (mRNA prime/viral boost) (Figure 3E), antibody levels remained stable over the nine-month period. Boosting with mRNA-based vaccines after a primary series with viral-vectored vaccines showed a high antibody fold change at month 3 (*p* < 0.0001, Figure 3H) and remained durable at month 6 compared to homologous vaccination (Figure 3I). By month 9, there was no difference in the antibody fold change between the heterologous and homologous vaccination groups (Figure 3J).

## 4. Discussion

Real-world data on long-term vaccine efficacy among different bio-geographical populations are important for assessing current vaccination strategies and in informing pandemic preparedness efforts. We examined vaccine-induced antibody responses after booster immunization in previously vaccinated Ghanaian adults. We showed that plasma RBD-specific IgG antibodies against the Wuhan strain were markedly high 6 months after the booster shot, but IgG levels decreased to pre-booster levels at 9 months post-booster. In contrast, booster vaccination marginally increased IgG levels against the Delta and Omicron variants. Heterologous priming with the viral-vectored vaccine and a booster with the mRNA-based Pfizer vaccine was superior in increasing RBD-IgG levels at months 3 and 6 compared to homologous mRNA vaccination and heterologous mRNA-viral-vectored vaccination. Plasma antibody levels correlated positively with the number of previous vaccine doses, as individuals who had received at least two vaccine doses prior to their inclusion in this study displayed significantly higher IgG levels than those who had received only one dose prior.

Our findings of high SARS-CoV-2 seroprevalence among our cohort support the outcome of previous serosurveys that show high SARS-CoV-2 exposure in Ghana [11,21,22,23]. Although our data shows high seroprevalence against both RBD and N-antibodies pre-booster, N-specific antibody levels were stable during the period of sampling, suggesting reduced COVID-19 transmission within the population. A cursory look at the reported cases by the GHS shows that the beginning of our sampling time coincided with a decline in a transmission peak that was largely driven by the Omicron variant. Within this healthy cohort, a significant proportion of our participants had developed antibodies against the Delta and Omicron variants from natural infections before receiving the booster vaccination. We had previously shown that, compared to naturally exposed unvaccinated adults from the same region, vaccinated individuals sampled around the same time developed significant ACE-2 inhibitory binding antibodies against Wuhan, Beta, Delta, and Omicron variants [18]. The marginal increase in post-boost Omicron- and Delta-specific IgG levels we observed is in contrast with previous studies that showed an increase in binding and neutralizing responses against Delta and Omicron sub-lineages after booster vaccinations, albeit at reduced levels compared to the prototypical Wuhan strain [24,25,26,27,28]. It is plausible that the additional booster shots resulted in a significant antibody response within our cohort but the resulting responses waned quickly by month 3 post-boost, as several studies have shown a peak antibody response by days 14–21 and rapid waning of Omicron-specific antibodies [3,26,28]. The increased Wuhan variant-specific but reduced post-booster responses against Delta and Omicron suggest immune imprinting by vaccination with antigens targeting the Wuhan variant. At the time of the study, Ghana had experienced a COVID-19 peak largely driven by the Omicron variant. However, primary and booster vaccinations with either mRNA or viral-vectored vaccines were based on Wuhan variant antigens, which could bias the recall responses toward the Wuhan variant as documented in other studies where breakthrough infections or vaccination led to a recall of antigenically related and vaccine-related immune memory [29,30,31,32]. Delta and Omicron variants are antigenically distant from the Wuhan variant, with Omicron having about 30 mutations within the spike gene and the majority of the mutations occurring in the receptor binding domain of the spike protein [33,34].

Durability of Wuhan-specific antibodies was enhanced among participants who had received at least two vaccine doses before receiving the additional dose compared to one-dose vaccine recipients. However, we note that the majority of the participants with one prior dose mainly received the Janssen vaccine. Thus, we were unable in the present study to draw a direct causal effect between the number of previous doses and the magnitude of antibody responses post booster vaccination vis-a-vis the effects of the different vaccine platforms. We could not examine the relationship between number of vaccine doses and antibodies against Delta and Omicron variants because the majority of participants in the subgroup analysis had previously received two vaccine doses. Nonetheless, we noted a correlation between number of previous vaccine doses and pre-booster Omicron-specific IgG levels, but this was not found at the other months. In our cohort, we observe that antibody titers in homologous mRNA vaccination are markedly high before and after booster dose. Heterologous regimens produced significant increases in RBD-specific IgG three months post-vaccination, indicating that either approach can transiently enhance humoral immunity. Generally, mRNA vaccines administered as boosters resulted in relatively higher RBD-specific antibody responses compared to the viral-vectored vaccine boosters, irrespective of the primer vaccine platform However, individuals primed with adenoviral-vectored vaccines and boosted with an mRNA vaccine exhibited slower decay rates and sustained antibody titers through month 9 compared to mRNA prime and viral-vectored boost. This suggest that initial priming with a viral-vectored vaccine and mRNA boost generate a more functionally diverse memory B-cell pool, consistent with observations from studies in other populations in both developed and low–middle income countries [35,36,37,38]. This observation contrasts with our earlier findings within a subset of this cohort where priming with mRNA and heterologous boosting with viral-vectored vaccine led to more durable cellular responses than homologous boosting [39]. mRNA vaccines have been established to be more immunogenic and induce high antibody titers compared to viral-vectored vaccines [40,41,42]. Our findings extend the use of a heterologous vaccine regimen to increase vaccine coverage in regions with reduced access to vaccines such as in sub-Saharan Africa

Our study provides useful insights on the durability of vaccine-induced antibody responses after booster vaccination within an African population. However, it has some limitations, a major one being our inability to examine functional antibody quality in assays such as viral neutralization and ACE-2 binding inhibition. However, other studies have shown a correlation between antibody binding titers, viral neutralization titers and vaccine efficacy [43,44,45,46]. Nonetheless, several studies have reported neutralization titers against Omicron and Delta are significantly reduced compared to the prototypical Wuhan strain in both infections and vaccinations [47,48,49,50]. The present analysis was confined to receptor-binding domains (RBDs) of the ancestral Wuhan strain, Delta, and Omicron variants. More recently emerged Omicron sub-lineages, including XBB and JN.1, were not included, primarily due to limited sample availability and resource constraints at the time of the study. Given the substantial antigenic divergence and immune escape properties documented for these sub-lineages [51,52], future studies incorporating such variants will be essential to fully characterize antibody cross-reactivity and the durability of humoral responses within this population. Furthermore, we did not examine clinical incidence related to breakthrough infections following booster vaccination within our cohort. This would have provided additional insights on the protective effects of the induced antibody response. We were restricted in our analysis of heterologous versus homologous responses to only the Wuhan strain due to the small sample size in the sub-analysis for Delta and Omicron antibody responses. The participants were primarily young adults, from a small geographical location with a median age of 22–23 years, leading to a narrow age range that restricted our ability to evaluate age-related differences in vaccine-induced antibody responses. Therefore, these findings may not be generalizable to older populations, in whom immunosenescence and comorbidity profiles could differentially shape vaccine immunogenicity.

## 5. Conclusions

In conclusion, we show that additional booster doses enhance durability of RBD-IgG levels for 3–6 months in previously vaccinated individuals. Heterologous vaccination priming with viral-vectored vaccine followed by an mRNA-based booster induced persistently high vaccine-specific responses at 6 months post-booster compared to homologous mRNA vaccination. This finding is particularly beneficial for vaccination efforts in many regions, especially in sub-Saharan Africa, where a consistent supply of the COVID-19 vaccine is challenging. Consequently, individuals are likely to receive COVID-19 vaccines developed on different platforms whenever they receive a booster shot.

## Figures and Tables

**Table 1:** Demographic and vaccination information study cohorts.

Variable	Total	Pfizer	Janssen	*p*-Value
*n*	181	91	90	
Age, yr median (range)	18–68 (18–68)	23 (18–68)	22 (18–65)	
Sex *n* (%)				
Female	66 (36.2)	29 (43.9)	37 (56.1)	*p* = 0.22
Male	115 (63.8)	62 (53.9)	53 (46.1)	
Booster regimen, *n* (%)				
Homologous prime-boost	115 (63.8)	45(39.1)	70 (60.9)	*p* < 0.0001
Heterologous prime-boost	66 (36.2)	46 (69.7)	20 (30.3)	
Number of previous vaccine doses *n* (%)				
1	50 (27.6)	37 (74)	13(26)	*p* < 0.0001
2	115 (63.5)	51 (44.3)	64 (55.7)	
3	16 (8.8)	3 (18.8)	13 (81.2)	

## Data Availability

Data supporting the findings of this study are available upon reasonable request.
